# The Influences of COVID-19 on Patients With Chronic Kidney Disease: A Multicenter Cross-Sectional Study

**DOI:** 10.3389/fpsyt.2021.754310

**Published:** 2021-11-25

**Authors:** Zheng Jiang, Jiang Liu, Lei Geng, Zhengxia Zhong, Jiaxing Tan, Dongmei Wen, Ling Zhou, Yi Tang, Wei Qin

**Affiliations:** ^1^West China School of Medicine, Sichuan University, Chengdu, China; ^2^Department of Nephrology, The Affiliated Hospital of Southwest Medical University, Luzhou, China; ^3^Sichuan Clinical Research Center for Nephropathy, Luzhou, China; ^4^Division of Nephrology, Department of Medicine, Affiliated Hospital of Zunyi Medical University, Zunyi, China; ^5^Department of Nephrology, People's Hospital of Jianyang, Chengdu, China; ^6^Division of Nephrology, Zigong Third People's Hospital, Zigong, China; ^7^Division of Nephrology, Department of Medicine, West China Hospital of Sichuan University, Chengdu, China

**Keywords:** COVID-19, chronic kidney disease, self-rating anxiety scale (SAS), psychological response, online consultation

## Abstract

**Background:** The outbreak of coronavirus disease 2019 (COVID-19) has attracted global attention. During the lockdown period of COVID-19, follow-up of many patients with chronic disease had been interrupted, which brought severe challenges to better management of their disease. This study aimed at exploring the change of illness, daily life, and psychological responses during the COVID-19 pandemic among chronic kidney disease (CKD) patients.

**Methods:** A total of 612 patients were enrolled in this study; 282 patients were categorized into the CKD stage 1–2 group and 330 patients were categorized into the CKD stage 3–5 group. Among two groups, 168 (27.5%) and 177 (28.9%) patients were female with a median age of 42 and 45, respectively. The study was conducted by collecting the questionnaires in five nephrology centers. The questionnaire consisted of assessment of anxiety by using the Self-Rating Anxiety Scale and the influences of COVID-19, which included basic demographic data, the influences of COVID-19 on illness and daily life, as well as the patients' psychological responses during the epidemic.

**Results:** A total of 612 patients were included and divided into two groups according to eGFR. Ninety-six patients (34%) in the CKD stage 1–2 group and 141 patients (42.7%) in the CKD stage 3–5 group had reduced their follow-up frequency (*p* = 0.031). More patients with CKD stages 1–2 consulted online (25.9%), *p* = 0.005. Besides, patients in the CKD stage 3–5 group tended to be more anxious about follow-up (*p* = 0.002), fearful of being infected with COVID-19 (*p* = 0.009), and more likely to feel symptoms getting worse (*p* = 0.006). The standard scores of SAS were 48.58 ± 7.082 and 51.19 ± 5.944 in the CKD stage 1–2 group and the CKD stage 3–5 group, respectively (*p* < 0.001). There were significant differences in the severity of anxiety (*p* = 0.004).

**Conclusion:** COVID-19 had a greater impact on patients with CKD stages 3–5 than those with stages 1–2 in terms of illness, daily life, and psychological disorder. Patients with CKD stages 3–5 were more anxious during the COVID-19 pandemic.

## Introduction

Since December 2019, the outbreak of coronavirus disease 2019 (COVID-19) has attracted global attention (1, 2). In China and many other countries, governments have implemented several compulsory measures, such as quarantine, restriction of mass gatherings and events, business and school closures, and reduced frequency of transport, to mitigate the spread of COVID-19 (3, 4).

It is reported that chronic kidney disease (CKD) patients are more vulnerable to COVID-19 than the general population (5). COVID-19 infection could lead to high frequency of renal abnormalities, which not only included massive proteinuria and hematuria, but also elevated serum creatinine and blood urea nitrogen (6). What is more, CKD is associated with an increased risk of pneumonia, and the pneumonia-related mortality rate in CKD patients seems to be 14–16 times higher than in the general population (7). During the lockdown period, owing to the lack of personal protective equipment and limited transport, COVID-19 seems to pose a threat to the health of CKD patients in terms of follow-up and acquiring drugs. They are more anxious about their illness and whether they could use vaccines against COVID-19. Due to these factors, CKD patients are more likely to gain negative emotions. A latest study demonstrated that hemodialysis patients had more severe trauma-related stress symptoms than peritoneal dialysis patients (8).

However, there were few studies focused on CKD patients who did not enter the maintenance dialysis stage. The impact of COVID-19 on different stages of CKD patients and the potential problems remained unknown. This study aimed at analyzing the change of illness, daily life, and psychological responses during the COVID-19 pandemic among CKD patients.

## Materials and Methods

### Study Design and Data Collection

This study was performed in five nephrology centers (West China Hospital, Affiliated Hospital of Southwest Medical University, Affiliated Hospital of Zunyi Medical University, People's Hospital of Jianyang city, and Zigong Third People's Hospital) from June to August 2020. The inclusion criteria were as follows: (1) CKD patients without dialysis over 14 years of age; (2) the patients could communicate smoothly and use smartphones independently or with the help of their families to perform the questionnaires. Those who could not use smartphones, did not finish all questions, or were unwilling to answer the questionnaire were excluded. Informed consent was obtained before the data collection. By scanning a Quick Response code on WeChat, the patients could enter the Wenjuanxing platform to complete the questionnaire in the outpatient department of five study centers and our WeChat follow-up group of CKD patients. The study was in compliance with the Declaration of Helsinki and was approved by the ethical committee of West China Hospital of Sichuan University.

### Composition of Questionnaire

The questionnaire consisted of two main parts: the influences of COVID-19 and assessment of anxiety. The first part included (1) basic demographic data; (2) the influences of COVID-19 on the illness and daily life; (3) and their psychological responses during the epidemic. The impact of COVID-19 on patients was measured by scores according to different degrees, ranging from 0 to 10. (1) Basic demographic data included age, sex, marital status, education level, and primary disease (Table 1). (2) Influences of COVID-19 on the illness and daily life included the severity of clinical symptoms and signs (edema, fatigue, poor appetite, dizziness, joint pain, rash, foam urine, hematuria, and blood pressure), non-COVID-19 infection, hospitalization for disease relapse, the frequency of follow-up, online consultation (including telephone hotline or smartphone application), the frequency of outside activities, and awareness of daily protection (Table 2). (3) Psychological responses during the epidemic included the attitudes toward follow-up and COVID-19 and the demand for psychological help (Table 3).

**Table 1:** Comparisons between demographic characteristics.

**Characteristics**	**CKD stages 1, 2**	**CKD stages 3–5**	***p*-value**
	***N* = 282**	***N* = 330**	
Female, *n* (%)	168 (27.5)	177 (28.9)	0.142
Age (years)	42 (35–51)	45 (35–56)	0.077
Marital status, *n* (%)^**^			<0.001
Married	207 (33.8)	315 (51.5)	
Unmarried	75 (12.3)	15 (2.5)	
Education level, *n* (%)^**^			<0.001
Primary	18 (2.9)	73 (11.9)	
Junior	59 (9.6)	90 (14.7)	
Senior	74 (12.1)	75 (12.3)	
University	131 (21.4)	92 (15)	
Disease, *n* (%)^**^	141 (23)	72 (11.8)	<0.001
CGN	53 (8.7)	52 (8.5)	
NS	4 (0.7)	46 (7.5)	
Metabolic related disease	47 (7.7)	58 (9.5)	
Autoimmune disease	37 (6)	102 (16.7)	
CRF or others			

F, female; CGN, chronic glomerulonephritis; NS, nephrotic syndrome; CRF, chronic renal failure. *p < 0.05,

** *p ≤ 0.01*.

**Table 2:** Comparisons of illness and daily life during the COVID-19 pandemic.

**Variables**	**CKD stages 1, 2**	**CKD stages 3–5**	***p*-value**
Non-COVID-19 infection, *n* (%)			0.905
No	244 (86.5)	287 (87)	
Yes	38 (13.5)	43 (13)	
Decreased frequency of follow-up, *n* (%)^*^			0.031
No	186 (66)	189 (57.3)	
Yes	96 (34)	141 (42.7)	
Online consultation, *n* (%)^**^			0.005
No	209 (74.1)	276 (83.6)	
Yes	73 (25.9)	54 (16.4)	
Hospitalization for disease relapse, *n* (%)			0.223
No	200 (70.9)	218 (66.1)	
Yes	82 (29.1)	112 (33.9)	
Aggravation of symptoms, *n* (%)^**^			0.006
No	215 (76.2)	217 (65.8)	
Yes	67 (23.8)	113 (34.2)	
Daily protection, *n* (%)			0.762
No	6 (2.1)	5 (1.5)	
Yes	276 (97.9)	325 (98.5)	
Frequency of follow-up, *n* (%)			0.928
0	52 (18.4)	60 (18.2)	
Once a month	113 (40.1)	127 (38.5)	
Twice a month	14 (5)	18 (5.5)	
Every 1 or 2 month	86 (30.5)	109 (33)	
More than 3 times a month	17 (6)	16 (4.8)	
Frequency of outside activities, *n* (%)			0.997
Nearly 0/week	130 (46.1)	149 (45.2)	
<3/week	84 (29.8)	100 (30.3)	
4–7/week	44 (15.6)	53 (16.1)	
>7/week	24 (8.5)	28 (8.4)	

* *p < 0.05*,

** *p ≤ 0.01*.

**Table 3:** Comparisons of psychological influences toward COVID-19.

**Variables**	**CKD stages 1, 2**	**CKD stages 3–5**	***p*-value**
Anxiety about follow-up, *n* (%)^**^			0.002
No	166 (58.9)	151 (45.8)	
Yes	116 (41.1)	179 (54.2)	
Fear of being infected with COVID-19, *n* (%)^**^			0.009
No	93 (33)	77 (23.3)	
Yes	189 (67)	253 (76.7)	
Avoiding social events, *n* (%)			0.848
No	216 (76.6)	255 (77.3)	
Yes	66 (23.4)	75 (22.7)	
Demand for psychological help, *n* (%)			0.633
No	247 (87.6)	284 (86.1)	
Yes	35 (12.4)	46 (13.9)	
Help from medical staff, *n* (%)^*^			0.038
No	41 (14.5)	30 (9.1)	
Yes	241 (85.5)	300 (90.9)	
Confidence in overcoming COVID-19, *n* (%)			0.199
No	7 (2.5)	3 (0.9)	
Yes	275 (97.5)	327 (99.1)	
Impact of COVID-19 on themselves (scores) ^*^	4.79 ± 2.64	5.25 ± 2.36	0.024

* *p < 0.05*,

** *p ≤ 0.01*.

### Self-Rating Anxiety Scale

The second part of the questionnaire was the Self-Rating Anxiety Scale (SAS), a self-report scale developed by Zung (9), which was used to specifically measure anxiety symptoms. The questionnaire had 20 self-report questions and scored on a four-point Likert scale, which was according to the frequency of symptoms, ranging from 1 to 4. The standard cutoff scores were used to define the following: ≤ 50 as no anxiety; 50–59 as minimal to mild anxiety; 60–69 as moderate anxiety; and ≥70 scores as severe anxiety. The SAS (Chinese version) had been widely used and demonstrated adequate reliability and validity (10).

### Statistical Analyses

Continuous variables were expressed as means ± SDs or medians (interquartile ranges). Categorical variables were expressed as number and percentages (%). Student's *t*-test or Mann–Whitney *U* test was used for continuous variables and χ^2^ test was used for categorical variables. The linear regression was used to examine the relationship between SAS scores and other variables. Then, the significant factors were further analyzed for SAS scores using multiple linear stepwise regression analysis. A two-tailed *p* < 0.05 was considered statistically significant. All statistical analysis was performed by using IBM SPSS statistics 26.0 software.

## Results

### Demographic Characteristics

In this study, 632 patients responded to the survey, and after removing 20 questionnaires for repeated or incomplete information, 612 patients were included. No patients were infected with COVID-19. Among them, 282 patients were categorized into the CKD stage 1–2 group and 330 patients were in the CKD stage 3–5 group.

There were no significant differences in sex and age between the two groups (Table 1). More patients with CKD stages 3–5 were married [207 patients (33.8%) in the CKD stage 1–2 group vs. 315 patients (51.5%) in the CKD stage 3–5 group, *p* < 0.001]. Patients with CKD stages 1–2 had a higher proportion of university degree (21.3%) and higher proportion of junior degree (14.7%) than in the group with CKD stages 3–5 (*p* < 0.001). The disease types of most patients with CKD stages 1–2 and stages 3–5 were chronic glomerulonephritis and chronic renal failure, respectively (*p* < 0.001, Table 1).

### Comparisons of Illness and Daily Life Toward COVID-19

In this study, the frequency of follow-up during the pandemic did not show differences between two groups. However, it was notable that the difference in decreased frequency of follow-up was significant. Ninety-six patients (34%) in the CKD stage 1–2 group and 141 patients (42.7%) in the CKD stage 3–5 group had reduced their follow-up frequency (*p* = 0.031). It could be noticed that more patients with CKD stages 1–2 consulted online (25.9%, *p* = 0.005). Besides, more patients in the CKD stage 3–5 group tended to feel their symptoms getting worse (*p* = 0.006). There were also no significant differences in non-COVID-19 infection, awareness of daily protection, hospitalization for disease relapse, and the frequency of outside activities (Table 2).

### Comparisons of Psychological Influences Toward COVID-19

Patients in the CKD stage 3–5 group felt more anxious about follow-up (*p* = 0.002) and more afraid of being infected with COVID-19 (*p* = 0.009). A total of 241 patients (85.5%) in the CKD stage 1–2 group and 300 patients (90.9%) in the CKD stage 3–5 group thought they had gained help from medical staff during the pandemic period (*p* = 0.038). The results of avoiding social events, demand for psychological help, confidence in overcoming COVID-19, and the impact of COVID-19 on themselves did not show differences between two groups (Table 3).

### Comparisons of SAS Scores and the Severity of Anxiety

The standard scores of SAS were 48.58 ± 7.082 and 51.19 ± 5.944 in the CKD stage 1–2 group and the CKD stage 3–5 group, respectively (*p* < 0.001). There were significant differences in the severity of anxiety; about 36.2% of patients in the CKD stage 1–2 group and 49.4% of patients in the CKD stage 3–5 group had symptoms of anxiety, most of which were mild (*p* = 0.004) (Table 4).

**Table 4:** Comparisons of SAS.

**Variables**	**CKD stages 1, 2**	**CKD stages 3–5**	***p*-value**
Standard scores^**^	48.58 ± 7.082	51.19 ± 5.944	<0.001
Severity^**^			0.004
None	180 (63.8)	167 (50.6)	
Mild	87 (30.9)	132 (40)	
Moderate	13 (4.6)	29 (8.8)	
Severe	2 (0.7)	2 (0.6)	

^*^p < 0.05,

** *p ≤ 0.01*.

### Univariate Analysis and Multivariate Analysis: Risk of SAS Scores

In the results of univariate analysis (Table 5), marital status (*p* = 0.015), university (*p* = 0.029), non-COVID-19 infection (*p* = 0.045), metabolic-related disease (*p* = 0.021), decreased frequency of follow-up (*p* < 0.001), hospitalization for disease relapse (*p* < 0.001), aggravation of symptoms (*p* < 0.001), anxiety about follow-up (*p* < 0.001), fear of being infected with COVID-19 (*p* < 0.001), avoiding social events (*p* = 0.011), demand for psychological help (*p* < 0.001), CKD stages (*p* < 0.001), the impact of COVID-19 on themselves (*p* < 0.001), the frequency of follow-up (*p* = 0.001), and the frequency of outside activities (*p* = 0.005) were chosen for the multiple linear stepwise regression model (Table 5). However, the multivariate analysis indicated that anxiety about follow-up (*p* = 0.005), demand for psychological help (*p* < 0.001), aggravation of symptoms (*p* < 0.001), fear of being infected with COVID-19 (*p* = 0.021), CKD stages (*p* = 0.001), the impact of COVID-19 on themselves (*p* < 0.001), university degree (*p* = 0.042), the frequency of follow-up (*p* = 0.007), and frequency of outside activities (*p* = 0.028) were associated with the severity of SAS independently (Table 6).

**Table 5:** Univariate analysis of SAS.

**Variables**	**Unstandardized coefficients**		**Standardized coefficients**	***p*-value**
	**B**	**SE**	**beta**	
Sex (male/female)	−0.46	0.54	−0.034	0.394
Age (years)	0.023	0.02	0.046	0.251
Marital status (married/unmarried)^*^	1.842	0.752	0.099	0.015
Education level				
Junior/primary	0.087	0.875	0.006	0.921
Senior/primary	0.003	0.875	0	0.998
University/primary^*^	−1.79	0.818	−0.013	0.029
Disease				
NS/CGN	1.507	0.787	0.086	0.056
Metabolic-related disease/CGN^*^	2.392	1.037	0.099	0.021
Autoimmune disease/CGN	0.864	0.787	0.049	0.273
CRF or others/CGN	0.783	0.72	0.05	0.277
Non-COVID-19-related infections (Y/N)^*^	1.579	0.787	0.081	0.045
Decreased frequency of follow-up (Y/N)^**^	2.232	0.542	0.164	<0.001
Online consultation (Y/N)	−0.456	0.66	−0.028	0.489
Hospitalization for disease relapse (Y/N)^**^	2.358	0.567	0.166	<0.001
Aggravation of symptoms (Y/N)^**^	3.215	0.573	0.222	<0.001
Anxiety about follow-up (Y/N)^**^	3.19	0.52	0.241	<0.001
Fear of being infected with COVID-19 (Y/N)^**^	2.64	0.588	0.179	<0.001
Avoiding social events (Y/N)^*^	1.617	0.632	0.103	0.011
Demand for psychological help (Y/N)^**^	3.66	0.619	0.233	<0.001
Help from medical staff (Y/N)	−0.367	0.438	−0.034	0.403
Awareness of daily protection (Y/N)	−0.207	1.506	−0.006	0.891
Confidence in overcoming COVID-19 (Y/N)	−2.935	2.108	−0.056	0.164
CKD stages (stages 3–5/stages 1, 2)^**^	2.616	0.527	0.197	<0.001
Impact of COVID-19 on themselves (scores)^**^	2.758	0.536	0.204	<0.001
Frequency of follow-up^**^	0.711	0.213	0.134	0.001
Frequency of outsides activities^**^	−0.766	0.275	−0.112	0.005

*N, no; Y, yes; CGN, chronic glomerulonephritis; NS, nephrotic syndrome; CRF, chronic renal failure*.

* *p < 0.05*,

** *p ≤ 0.01*.

**Table 6:** Multivariate analysis of SAS.

**Variables**	**Unstandardized coefficients**		**Standardized coefficients**	***p*-value**	**VIF**
	**B**	**SE**	**Beta**		
Anxiety about follow-up (Y/N)^**^	1.453	0.512	0.11	0.005	1.01
Demand for psychological help (Y/N)^**^	4.553	0.704	0.233	<0.001	1.161
Aggravation of symptoms (Y/N)^**^	2.243	0.534	0.155	<0.001	1.05
Fear of being infected with COVID-19 (Y/N)^*^	1.289	0.558	0.087	0.021	1.108
CKD stages (stages 3–5/stages 1, 2)^**^	1.628	0.494	0.123	0.001	1.076
Impact of COVID-19 on themselves (scores)^**^	0.366	0.099	0.138	<0.001	1.076
Frequency of follow-up^**^	0.525	0.194	0.099	0.007	1.031
Frequency of outside activities^*^	−0.554	0.252	−0.081	0.028	1.058
University/primary^*^	−1.051	0.516	−0.076	0.042	1.091

*N, no; Y, yes*.

* *p < 0.05*,

** *p ≤ 0.01*.

## Discussion

In this study, we found that COVID-19 had a greater impact on patients with CKD stages 3–5 than on those with CKD stages 1–2 in terms of illness, daily life, and psychological disorder. In addition, patients with CKD stages 3–5 seemed more anxious. The COVID-19 pandemic has a strong impact on the lives and work of people all over the world. The whole society is under great pressure for unemployment, infection, being separated from family, death, and so on (11–13). For CKD patients, they often need regular follow-up in the hospital. However, during the lockdown period, they cannot visit the hospital on time, which brings difficulties in better controlling their disease and the adjustment of treatment. The epidemic poses a huge challenge to patients with CKD because they require frequent care and support, and these needs are still required during the pandemic (5). Some researchers had realized the unique challenges that kidney disease patients experienced during the COVID-19 pandemic. These patients may be at increased risk of infection or worse outcomes and were already facing obstacles in their routine medical care (14, 15).

In this study, we investigated the daily life, illness, and psychological responses of CKD patients during the COVID-19 pandemic. We found that there were significant differences in online consultation, aggravation of symptoms, fear of being infected with COVID-19, decreased frequency of follow-up, anxiety about follow-up, the impact of COVID-19 on themselves by self-scoring, and the help from medical staff between patients with CKD stages 1–2 and stages 3–5. We were surprised to observe that the median scores of SAS were not high in the whole included patients. The median scores of patients with CKD stages 1–2 and CKD stages 3–5 were 48.58 and 51.19, which represented normal and mild anxiety, respectively. Overall, most CKD patients could calmly handle the situation. Patients with CKD stages 3–5 seemed to be more anxious during this period.

Several reasons might lead to these differences between the two groups. The condition of patients with CKD stages 1–2 was relatively stable with simpler drug treatments, fewer complications, and slower progression of disease. However, the condition of patients with CKD stages 3–5 was more serious, with complications in most cases, and that was why they needed to visit the hospital more often. Owing to the usual higher frequency of follow-up of the CKD stage 3–5 patients, their decreased frequency of follow-up would be more apparent during the lockdown period.

During the epidemic, hospitals opened a special COVID-19 telephone hotline and smartphone application for online consultations, through which medical staff offered suggestions and interventions out of the hospital (16). It could reduce crowd gathering in offline hospitals (17). Especially for those who had mild symptoms, online doctors could give professional advice on self-management and treatment (18). Many hospitals began to offer internet-based drug prescription and delivery service for patients with common and chronic diseases (16). However, the illness of patients with CKD stages 3–5 tended to be more severe and some drugs could not be obtained online; for example, erythropoietin and insulin must be transported in low temperature, which was difficult to carry out. CKD patients with anemia or diabetes may not have good access to the needed drugs, which proved harmful to their condition. Furthermore, some special examination items could only be performed in the central hospitals, which could not be solved by online consultation. Consequently, it could not take the place of offline treatment completely. Also, patients with CKD stages 1–2 had a higher education level, which may make them more accustomed to using telephones for the new type of treatment. These were reasons why patients with CKD stages 3–5 had a lower proportion of online consultation. Better drug delivery system for some special drugs and improvements in more convenient ways to online access could contribute to the popularity of online consultation. A future area community lab center would be beneficial to patients with chronic disease for blood or other examination during an epidemic.

Besides, because of the lack of enough protective equipment, transportation inconvenience, and fear of being infected in the hospital, which was a high-risk area full of sick patients (19), patients with CKD stages 3–5 could be more anxious about follow-up in the hospital. Additionally, due to the more severe illness of these patients, they could easily feel their symptoms getting worse.

SAS was a norm-referenced screener with adequate reliability and validity, which had been shown to discriminate anxiety from mood disorders (20). All the reasons mentioned above may contribute to the higher scores of SAS in patients with CKD stages 3–5. The results of multivariate analysis indicated that CKD stage was one of the independent risk factors of SAS. Despite the fact that the impact of COVID-19 on patients themselves and the demand for psychological help had no statistical difference between the two groups, these factors were all positively associated with SAS. Education level and frequency of outside activities were negatively associated with SAS, which showed that patients with a university degree or higher frequency of outside activities were less likely to be anxious. Patients with a higher education level may know more proper ways of self-protection, which is helpful in facing the epidemic more calmly and rationally. In addition, patients had a higher frequency of outside activities mainly because they need better work, which often made them more adaptable in the society coexisting with the epidemic.

Actually, in some previous studies, researchers had realized that psychiatric disorders were common among the public during the 2003 SARS and 2014 Ebola virus outbreaks or other epidemics (21, 22). In 2016, it was reported that during the Middle East Respiratory Syndrome (MERS) pandemic, about 47.2% MERS patients had symptoms of anxiety and 52.8% had feelings of anger (23). Additionally, many studies concentrated on infected patients, the general population (24), frontline health and social care professionals (25), and students (26), and only few studies focused on patients with chronic disease. This study was the first to report on how CKD patients without dialysis reacted during the pandemic. To date, there are still many confirmed cases of COVID-19 worldwide. Our study might contribute to improving the management and treatment of CKD patients during the pandemic. Careful psychological assessment and sufficient mental support should be provided to more CKD patients, especially those with lower eGFR.

However, there were some limitations in our study. First, our data were collected in Sichuan province, which was not a high prevalence area. Second, there were no data about how CKD patients obtained drugs during the lockdown period. Third, some parts of the questionnaire were answered according to the patients' subjective perception, which may lack objectivity.

## Conclusion

In conclusion, compared with patients with CKD stages 1–2, patients with CKD stages 3–5 were more affected in terms of illness, daily life, and psychological disorder during the COVID-19 pandemic. They seemed more anxious when confronted with such infectious diseases. More careful management of illness and mental support should be provided to CKD patients, especially those with lower eGFR.

## Data Availability Statement

The raw data supporting the conclusions of this article will be made available by the authors, without undue reservation.

## Ethics Statement

The studies involving human participants were reviewed and approved by the Ethical Committee of West China Hospital of Sichuan University. The patients/participants provided their written informed consent to participate in this study. Written informed consent was obtained from the individual(s) for the publication of any potentially identifiable images or data included in this article.

## Author Contributions

ZJ designed the study, collected data, performed statistical analyses, and wrote the manuscript. JL, LG, ZZ, DW, and LZ collected the data. JT collected the data and performed statistical analyses. WQ and YT designed the study and critically revised the work. All authors read and approved the final version of the manuscript.

## Conflict of Interest

The authors declare that the research was conducted in the absence of any commercial or financial relationships that could be construed as a potential conflict of interest.

## Publisher's Note

All claims expressed in this article are solely those of the authors and do not necessarily represent those of their affiliated organizations, or those of the publisher, the editors and the reviewers. Any product that may be evaluated in this article, or claim that may be made by its manufacturer, is not guaranteed or endorsed by the publisher.
